# Emerging Progress in Nausea and Vomiting of Pregnancy and Hyperemesis Gravidarum: Challenges and Opportunities

**DOI:** 10.3389/fmed.2021.809270

**Published:** 2022-01-10

**Authors:** Chuan Liu, Guo Zhao, Danni Qiao, Lintao Wang, Yeling He, Mingge Zhao, Yuanyuan Fan, Enshe Jiang

**Affiliations:** ^1^School of Medicine, Henan University, Kaifeng, China; ^2^Department of Neurology, The First Affiliated Hospital of Henan University, Kaifeng, China; ^3^School of Life Sciences, Henan University, Kaifeng, China; ^4^Institute of Nursing and Health, School of Nursing and Health, Henan University, Kaifeng, China; ^5^Henan International Joint Laboratory for Nuclear Protein Regulation, Henan University, Kaifeng, China

**Keywords:** nausea and vomiting of pregnancy, hyperemesis gravidarum, pathophysiology, growth/differentiation factor 15, management

## Abstract

Nausea and vomiting of pregnancy (NVP) is a common condition that affects up to 70% of pregnant women. Hyperemesis gravidarum (HG) is considered the serious form of NVP, which is reported in 0.3–10.8% of pregnant women. NVP has a relatively benign course, but HG can be linked with some poor maternal, fetal, and offspring outcomes. The exact causes of NVP and HG are unknown, but various factors have been hypothesized to be associated with pathogenesis. With the advance of precision medicine and molecular biology, some genetic factors such as growth/differentiation factor 15 (GDF15) have become therapeutic targets. In our review, we summarize the historical hypotheses of the pathogenesis of NVP and HG including hormonal factors, *Helicobacter pylori*, gastrointestinal dysmotility, placenta-related factors, psychosocial factors, and new factors identified by genetics. We also highlight some approaches to the management of NVP and HG, including pharmacological treatment, complementary treatment, and some supporting treatments. Looking to the future, progress in understanding NVP and HG may reduce the adverse outcomes and improve the maternal quality of life during pregnancy.

## Introduction

Nausea and vomiting of pregnancy (NVP) is a common condition that affects up to 70% of pregnant women (1). NVP is common and usually begins at 6–8 weeks of gestation and generally resolves by 16–20 weeks (2). Hyperemesis gravidarum (HG) is generally considered to be the most serious expression of NVP, and is reported in 0.3 to 10.8% of pregnant women (1, 3). Ethnic differences in HG incidence have been supported by many population studies (4). Some researchers reveal that NVP is more common in western countries and urban populations, and is rare among Africans, Native Americans, Inuit, and most Asian populations (5). In a multivariate analysis designed to control for confounders (*n* = 367 women), the researchers noted a lower incidence among black and Asian women (6). Similarly, Bashiri et al. showed that Jews have a higher incidence of HG than the Bedouin (7). However, other researchers came to different conclusions. For example, Bailit et al. conducted a study with 520,739 newborns in California that reported a 0.5% incidence of maternal HG. In this California population, non-white and non-Hispanic patients had higher rates of HG hospitalization than their white counterparts (4). A New Zealand study reported that people of European origin had similar levels of HG (2%), but Pacific Island women had much higher levels, with their HG levels up to 4-fold higher (8). In northern Israel, a small study found an incidence rate of 1.2% among Arab and Jewish women (9).

NVP is an extremely common pregnancy disorder that ranges from mild to moderate; severe nausea and vomiting are the second most common indications for pregnancy hospitalization, and are considered as pathological HG (10, 11). Symptoms of NVP usually peak between 10 and 16 weeks of pregnancy and usually disappear on their own (12). NVP is erroneously called “morning sickness” as only 1.8 percent of women report nausea only in the morning, while 80% report nausea throughout the day. Researchers also described an episodic pattern of NVP, with 95.2% of women presenting symptoms before and after midday (12, 13). In a meta-analysis to quantify global rates, Einarson et al. found that the reported rates of pregnant women experiencing NVP varied widely. They also reported that of their cohort of women with NVP symptoms, the severity was rated as mild in 40%, moderate in 46%, and severe in 14%, while HG prevalence is typically 1.1% (14). Some researchers noted that the incidence of NVP was 40% higher in women under 20 years of age, first-time mothers, women with <12 years of education, non-smokers, and obese women. Some inconsistent reasons, such as family income, parity, and oral contraceptive use before pregnancy may also be associated with the degree of NVP in pregnant women (12, 15, 16). In addition, it has been reported that a higher incidence of NVP occurs during the first trimester of multiple pregnancies compared to women with a single pregnancy (12). Most studies have found that NVP is associated with good fetal outcomes. In a review of meta-analyses, researchers found that NVP was strongly associated with a reduced risk of miscarriage (17). However, NVP still has some adverse outcomes, such as an increased risk of intrauterine growth retardation in women with severe NVP (18). Similarly, women with severe NVP are at increased risk of low birth weight, possibly owing to the deleterious effects of nausea and vomiting on maternal nutrition (19). Hinkle et al. also reported an association between pregnancy loss and NVP (20).

HG occurs during the first trimester of pregnancy, usually starting at 4 or 5 weeks (11). In 1956, a panel appointed by the American Pharmaceutical and Chemical Boards first defined HG as refractory vomiting and a group of disorders including electrolyte balance changes, weight loss ≥5%, ketosis and ketonuria, neurological disorders, liver damage, retinal hemorrhage, and kidney damage. A recent international consensus definition for HG consists of: symptoms start in early pregnancy (before 16 weeks gestational age); nausea and vomiting, at least one of which is severe; inability to eat and/or drink normally; strongly limits daily living activities. Signs of dehydration were deemed contributory for the definition for HG (21). Clinical practice regards HG as the most severe expression of NVP, with complications such as dehydration or metabolic disorders (weight loss, electrolyte deficiency, or malnutrition) (1). Robinson et al. reported that maternal malnutrition owing to HG can lead to vitamin K deficiency, which can induce clotting (22). In addition, HG may be associated with many complications, including Wernicke encephalopathy (brain damage caused by vitamin B1 deficiency), acute liver and kidney failure, esophageal rupture, pneumothorax, preeclampsia, placental abruption, and neurodevelopmental delay of the fetus. Other adverse outcomes include preterm birth, small for gestational age, electrolyte disturbances which can lead to cardiac dysrhythmia, neuromuscular and renal complications, thyrotoxicosis, and maternal death (1, 23–26).

## Pathogenesis

### Hormonal Factors

#### GDF15-GFRAL Axis

Genes are risk factors for HG and NVP (27, 28) and recent advances involving familial aggregates and twins have demonstrated this role (27). Vikanes reported that the recurrence of HG can cross generations; if a mother has HG, her daughter's risk of HG is significantly increased by 3-fold (27). Having a sister with HG increases an individual's risk by 17-fold. Women with HG are reported to have the same proportion of maternal and paternal grandmothers with HG, suggesting the possibility that HG is passed down through the maternal and/or paternal lines (29). Conde et al. conducted a twin study where the results estimated the presence of NVP heritability at 73%, with variations in duration and severity >50% (28). Data from Norway's large twin population shows that identical twin women used more nausea medications during pregnancy than fraternal twins. In addition, women whose mothers experienced nausea during pregnancy reported higher levels of nausea (30).

With the advent of the era of precision medicine, we can more closely address pathogenesis at the molecular and gene levels. Fejzo et al. conducted a genome-wide association study (GWAS) which included 53,731 women of European descent to examine the molecular and gene etiology of HG. They reported two loci that reached genome-wide significance: chr19p13.11 and chr4q12, containing GDF15 (encoding growth/differentiation factor 15) and IGFBP7 (encoding insulin-like growth factor-binding protein 7) genes, respectively (31). Of the two, GDF15 has attracted the most attention from researchers.

**Figure 1:**
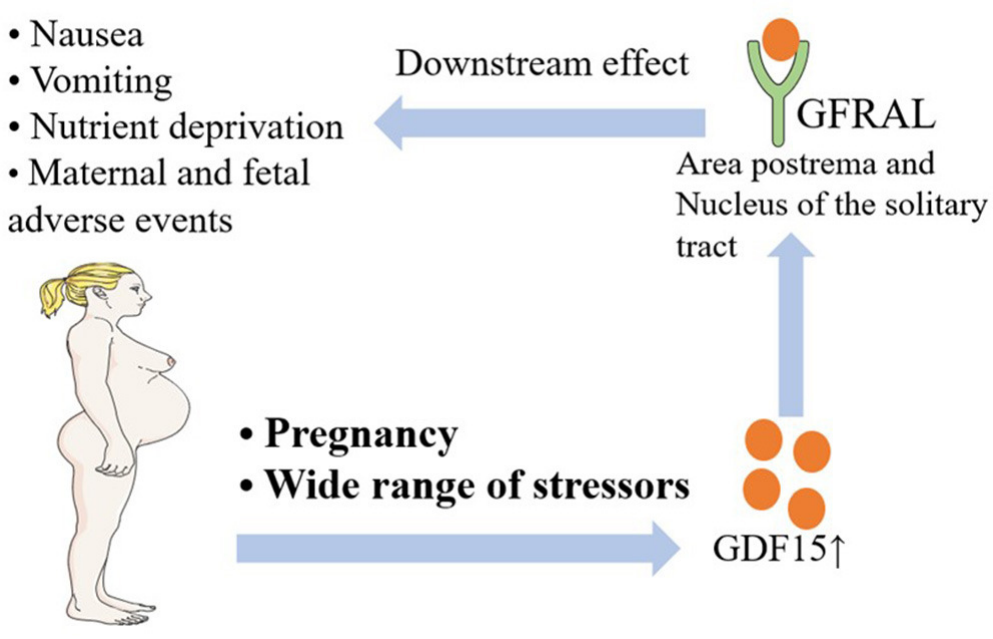
A brief introduction to the relationship between NVP and HG and the GDF15-GFRAL axis. NVP, Nausea and Vomiting of Pregnancy; HG, Hyperemesis Gravidarum; GDF15, Growth/Differentiation Factor 15; GFRAL, Glial-derived neurotrophic factor (GDNF) Family Receptor Alpha-Like.

GDF15 is a member in the Transforming Growth Factor-β (TGF-β) superfamily, which is secreted mostly by the placenta, prostate, and some abdominal viscera cells exposed to a wide range of stressors (32, 33). In February 2021, Zhang et al. identified the cell types in the area postrema of the brain that mediate nausea associated behaviors. A fourth cluster of excitatory neurons is the Glial-derived neurotrophic factor (GDNF) family receptor alpha-like (GFRAL), which is the receptor of GDF15 (34). Some researchers have already offered some perspectives regarding the GDF15-GFRAL axis and NVP or HG; these relationships are briefly introduced in Figure 1. Patel et al. showed that increased circulating GDF15 levels in nutritional stress might provide an aversive endocrine signal to the brain of both mice and humans (35). In addition, elevated circulating levels of GDF15 were correlated with the onset and progression of cachexia in mice and in patients with cancer, which causes symptoms similar to HG, such as nausea, weight loss, and muscle wasting (36, 37). Through the examination of blood samples from 791 pregnant women, Petry et al. found that circulating GDF15 concentrations were higher in women reporting vomiting in the second trimester than in women without pregnancy nausea or vomiting. Furthermore, the data showed that increased serum GDF15 levels were significantly associated with both second trimester vomiting and antiemetic use during pregnancy, supporting the concept that GDF15 might play a pathogenic role in pregnancy-associated vomiting (38). Similarly, Fejzo et al. showed that GDF15 serum levels were significantly increased in women hospitalized with HG, further supporting the role of GDF15 in the pathogenesis of HG (39). Furthermore, Fejzo et al. found that single nucleotide polymorphisms (SNPs) associated with higher levels of GDF15 segregated with disease in HG families, showing that GDF15 plays a role in the pathogenesis of both familial and recurrent cases of HG (40). GDF15 is a very promising intervention target for NVP and HG, and more studies are needed in the future to further promote the translational application of the GDF15- GFRAL axis.

The GDF15-GFRAL axis is currently considered by most researchers to be the most likely pathogenic mechanism of NVP and HG. However, since this has only recently been reported, related studies are still few in number. Larger and better designed studies should be performed to allow researchers and physicians to better understand the pathogenesis of NVP and HG and to develop more specific and effective drugs that will broadly improve maternal and fetal outcomes.

#### Human Chorionic Gonadotropin

Human chorionic gonadotropin (hCG) is a pregnancy hormone secreted by the placental cytotrophoblast cell layer that is related to fetal growth and various placental, uterine, and fetal functions (41). hCG has been widely considered to be an important factor in the pathogenesis of NVP and HG (42). This is mainly based on the peaks of hCG production and NVP symptoms, both of which occur in gestational weeks 12 and 14 (43, 44). Decades of research has already investigated the relationship between hCG and NVP or HG, we summarized the related studies below.

The hormone hCG is upregulated in early pregnancy at the same time that symptoms of NVP and HG occur (45). In 2014, Niemeijer et al. identified 18 studies demonstrating that elevated serum hCG levels were associated with NVP or HG, while 13 studies showed no relationship between hCG and NVP (42). Furthermore, Korevaar et al. noted a significant correlation between hCG and NVP symptoms in 8,195 women (46); however, another retrospective cohort study of 4,372 pregnancies found no evidence of such association (47). More recently, high hCG levels were also observed in 318 participants with NVP (48).

Elsewhere, serum hCG levels were significantly increased in HG during a prospective study performed on 167 first trimester women (49). Meanwhile, levels of beta-hCG have also been related to HG (50). In addition, serum T4 may also be related to high hCG, and serum TSH was negatively associated with high hCG in pregnancies with NVP (51). A further study conducted by Kauppila et al. showed that serum hCG concentrations were higher in the women with HG at 7–8 weeks in 42 patients with HG compared to 115 women with normal pregnancies (52).

Although many studies link hCG to NVP and HG, some studies also found no relationship between serum hCG in pregnant women during the first trimester and the frequency of NVP. In a study by Soules et al., even in a subset of women with molar pregnancies in whom levels of hCG were five to 10-fold higher than in controls, no correlation was found (53). Furthermore, researchers have found high levels of hCG to be associated with fetal growth retardation and preterm delivery, whereas NVP appears to be protective for preterm delivery (54), making it unlikely that hCG is the sole contributor to the pathogenesis of NVP. In addition, genetic studies have not found evidence to support an association with hCG nor its receptor. Two studies measured GDF15 and hCG levels and provided strong evidence of a role for GDF15 and strong evidence against a role for hCG. Fejzo et al. measured serum levels of both GDF15 and hCG and reported that serum GDF15 was notably increased in women with HG at 12 weeks of gestation. However, serum hCG was not significantly different between cases and controls (39). Petry et al. found a strong positive association between circulating GDF15 and hCG, and that serum GDF15 concentrations were positively associated with second trimester vomiting and maternal antiemetic use in pregnancy. However, hCG levels were not significantly higher in women reporting vomiting in the second trimester of pregnancy (38).

Therefore, it is time to pay greater attention to the molecular pathogenesis of NVP and HG, such as the impact of GDF15. More varied well-designed studies are needed instead of concentrating on the traditionally important hormone hCG.

#### Thyroid Hormone

Pregnancy is a high metabolic state because the fetus has an increased need for a healthy mother to meet its requirements for growth and development; to meet this need, the mother's thyroid function is usually increased (51). In approximately one-third of all cases of HG (*n* = 25), patients presented with transient biochemical evidence of thyrotoxicosis (55). So far, 12 studies suggest that adverse increased thyroid hormone production may be a cause of HG or NVP (51, 55–57); but three studies have suggested that hyperthyroidism may not be a cause of such symptoms (56–58).

In a case-control study, enzyme immunoassay was used to compare 30 patients with HG and 30 pregnant women without HG. Researchers found that levels of T4 and hCG increased simultaneously, while the level of TSH decreased with increasing hCG (51). Similarly, in a prospective study that included 54 women with HG and 42 women without HG, the results showed that serum levels of free T3 and free T4 in the HG group were significantly higher than those in the control group, but the level of TSH was significantly lower than the control. Numerous researchers have demonstrated a correlation between transient hyperthyroidism and HG. The main cause of transient hyperthyroidism may be the stimulating effect of hCG (59). The thyrotropic activity of hCG can be explained by the molecular homology between hCG and TSH and their receptors (59). However, with the lack of convincing evidence to support the hCG theory, more studies addressing the molecular mechanism of the effects of thyroid hormone on NVP and HG are needed.

A more recent study suggests that thyroid hormones induce the overexpression of RyR2, which encodes Ryanodine receptor 2, a stress-induced calcium channel associated with recurrent vomiting syndrome, which is the only ryanodine receptor expressed in the cerebral vomiting center and has been associated with vomiting signaling pathways in animal models (1). Fejzo et al. proposed that mutations in genes in the ryanodine receptor-signaling pathway may increase risk of HG, although follow-up studies are needed as the causal relationship has not been determined (60).

In addition, a separate trial that enrolled 134 hyperthyroid patients and 105 healthy subjects suggested that the level of GDF15 was significantly increased in hyperthyroid patients, and the expression of GDF15 in mice was up-regulated by thyroid hormone treatment. Therefore, thyroid dysfunction may play a role in NVP and HG by promoting elevated levels of GDF15 (61).

In a study of 10 pregnant women with HG, the results showed serum levels of total and free thyroid hormones, thyroid-stimulating hormone (TSH), thyroid-binding globulin (TBG), and hCG in HG patients were not significantly different from those in a normal pregnancy control group (57). Therefore, Wilson et al. suggested that thyroid hormone levels, thyroid antibodies, and hCG levels may not a cause of the vomiting (57).

In summary, only an association between T4 and hCG is known, and it is not clear whether there is a direct relationship between T4 and NVP or HG. Thyroid hormones may play a mediating role in many complex mechanisms, and further research is needed to unravel them.

#### Other Hormones

Some researchers regard TNF-α as a factor in the pathogenesis of NVP and HG. Increased concentrations of free DNA in fetal cells were found in the serum of pregnant women with HG, leading to an overactive immune response and trophoblast damage in the mother. It is speculated that an overactivated maternal immune system causes HG (62). CD4 positive TH cells can be divided into TH1 and TH2 cells according to their cytokine production mode. TH1 cells mainly synthesize interleukin (IL-2) and tumor necrosis factor (TNF-α) to induce cellular immunity. TH2 cells mainly produce IL-4, IL-5, IL-6, IL-10, and IL-13, and promote humoral immunity (63). Tumor necrosis factor-α (TNF-α) is associated with HG (11). However, some researchers present opposite opinions regarding the relationship between TNF-α and HG.

TNF-α is a protein released by macrophages that has a direct cytotoxic effect on tumor cells, stimulates immunoreactive cells, and induces cell proliferation and differentiation (64). In a prospective study that included 90 women, the researchers found that serum TNF-α levels were significantly higher in HG patients than in healthy and non-pregnant women (64). In Yoneyama's study, TNF-α levels were also found to be significantly higher in the HG group than in the non-pregnant and normal pregnancy groups (65).

In contrast, some researchers consider TNF-α as an inhibitor of NVP and HG. To investigate the direct effect of TNF-α on trophoblast cells, Ohashi et al. created placental cell models using nuclear chorionic cancer cell lines. The results showed that TNF-α inhibited the secretion of hCG in NUC1 cells at concentrations of 1–100 U/mL. At 1, 10, and 100 U/mL, TNF-α significantly reduced the level of hCG secretion at 24 h, which was 88, 81, and 71% of the control group, respectively. They concluded that TNF-α may act on trophoblast cells during early pregnancy to reduce hCG secretion (66).

However, Silen et al. found that TNF-α did not appear to be involved in regulating hCG production in human chorionic cancer cell lines, and they hypothesized that TNF-α had no independent effect on hCG production. Human chorionic cancer cell lines retain many of the characteristics of normal human trophoblast cells, including their ability to produce hCG, progesterone, and placental lactogen (67).

To date, it is not clear whether the high levels of TNF-α in HG patients are a cause of, or are caused by HG. However, because HG is a self-limiting disease, the elevation of these seemingly random immune factors may be part of a compensatory response that limits its progression (68). Additional studies are not likely to resolve these issues, and time and resources focusing on etiology would be better focused elsewhere.

Estradiol is considered to contribute to the pathogenesis of NVP. Lagiou et al. reported that estradiol levels were positively linked with NVP (69). Some researchers also observed that estradiol levels were increased in HG. Jordan et al. showed that an intolerance of oral contraceptives was closely linked with HG (70). Depue et al. showed that the total level of estradiol was 26% higher in an HG cohort than in the control group (71). Oruc et al. also reported that the concentration of estradiol in HG patients was significantly higher than in the control group (72). It is postulated that estradiol can relax smooth muscle and slow gastric emptying by increasing the production of nitric oxide through activating nitric oxide synthetase (11). However, a review including 17 studies found that only five showed a positive correlation between NVP and estrogen (73). Moreover, the peak times of estrogen and HG are not in concordance; NVP and HG peak in the first trimester (44), whereas estrogen levels peak in the third trimester (68). Given that a potential role for estrogen has been well-studied with conflicting results, it is unlikely to play a role in the etiology and resources should be diverted elsewhere.

Some researchers also considered progesterone as a contributor to NVP and HG. Using electrogastrography, Walsh et al. found that the gastric slow-wave rhythm of NVP can be induced in non-pregnant women by progesterone alone or with estradiol in doses that mimic the levels in pregnancy (74). Meanwhile Verberg et al. mentioned that some iatrogenic processes increased the levels of progesterone but did not produce an elevated incidence of HG; for example, pregnancies in which progesterone is administered for luteal phase support, or pregnancies with multiple corpora lutea caused by controlled ovarian stimulation, indicating that high levels of progesterone (endogenous or exogenous) alone may not cause HG (68). However, the progesterone receptor gene has been linked to HG in a genome-wide association study and replication study, which provides evidence the progesterone signaling pathway can be associated with HG without abnormal levels of progesterone (1, 31). More comparative and molecular studies should be conducted to identify the role of progesterone in NVP and HG.

### Helicobacter pylori

Recently, many studies have noted an underlying association between *Helicobacter pylori (H. pylori)* infection and the pathogenesis of HG and NVP (75, 76). Chronic *H. pylori* infection is a risk factor for HG and NVP even though it may not be the single cause of the disorder (77). Therefore, it is important to further study these underlying mechanisms.

Erdem et al. found that a majority of pregnant women with HG are seropositive for *H. pylori* infection, while there is no relationship between seropositivity for *H. pylori* infection and duration of HG symptoms (78). Studies based on firstly 274 pregnant Chilean women, and then 95 pregnant women with HG and 116 asymptomatic pregnant women, also came to the same conclusion (79, 80). However, Bagis revealed that the degree of gastric complaints of HG patients may be associated with the density of *H. pylori* infection based on 20 patients with severe HG and 10 pregnant women without gastric complaints (81). Additionally, there was also a remarkable association between groups with *H. pylori* seropositivity and frequency of vomiting in pregnant women (82). Furthermore, a lower socio-economic status might be a crucial risk factor for *H. pylori* infection in pregnant women with HG, based on 56 pregnant women with HG and 90 control pregnant women (83). Another study showed that *H. pylori* can increase the risk of HG with a dose-response pattern and is particularly strong in Africans based on 244 pregnant women with HG and 244 control pregnant women (84). In two meta-analyses, *H. pylori* has been shown to be related to an increased risk of NVP and HG in pregnant women (42, 85), and may be associated with some adverse symptoms, including reduced birth weight, low maternal weight gain, and small for gestational age (86). Importantly, Cevrioglu found that the *H. pylori* stool antigen (HpSA) test exhibits more efficient, realistic, and reliable results compared with specific Igs formed against *H. pylori* in pregnant women with HG (87). Similarly, *H. pylori* IgG and IgM levels in blood samples and the PCR identification of *H. pylori* DNA in saliva are also positive related (88).

Nevertheless, some studies found no correlation between NVP or HG and *H. pylori*. Most pregnant women who are seropositive for *H. pylori* do not have HG symptoms (89). Jacobson et al. found no relationship between HG and *H. pylori* seropositivity in 53 subjects and 153 controls (90). Meanwhile, *H. pylori* seropositivity had no association with gastrointestinal symptoms later in pregnancy (91). Beyazit et al. reported that HG is an oxidative stress condition, regardless of *H. Pylori* infection, based on women with pregnancies complicated by HG (*n* = 33), pregnant women without HG (*n* = 30), and healthy non-pregnant women (*n* = 31) (92). However, in 2018, Goymen et al. showed that HG may lead to a significantly increased oxidative burden and slightly decreased antioxidative capacity of saliva, which may be the result of *H pylori* infection and that such infection was more common in women with poor oral hygiene and HG (93).

In conclusion, *H. pylori* infection may exacerbate HG or NVP symptoms, but studies are unclear whether the eradication of *H. pylori* infection before pregnancy can significantly lower HG risk. Hence, studies are urgently needed to investigate this point.

### Gastrointestinal Dysmotility

Upper intestinal motility disorder has been hypothesized to be a cause of HG and NVP (94). Women with NVP may have a gastric slow wave rhythm (74, 95, 96). Changes of gastric slow wave rhythm during pregnancy may be caused by an increase in progesterone and estrogen levels (74). GDF15 has also been shown to delay gastric emptying which may contribute to nausea (1). Meanwhile, several slow-wave abnormalities are associated with functional motor disorders, such as gastroparesis, chronic unexplained nausea and vomiting, and functional dyspepsia (97). Potential extragastric related factors for nausea and vomiting include delayed distal gastric transport, autonomic nervous system abnormalities, altered central nervous system activation, metabolic disorders, and psychological dysfunction (98).

Gastric slow-wave rhythm disorder during pregnancy may be the comprehensive result of increased endogenous estrogen and progesterone levels (95). A study by Owyang et al. found that progesterone and estrogen may disrupt the slow wave rhythm of the stomach in susceptible individuals, resulting in nausea and vomiting in pregnant women (99). In addition, elevated progesterone levels can lead to muscle relaxation, which may be another cause of upper bowel dyskinesia (94).

Low esophageal sphincter function is affected by hormonal changes. It is manifest mainly through heartburn, but also by nausea and vomiting. In general, lower esophageal sphincter pressure is decreased during pregnancy. Van Thiel et al. mentioned that resting lower esophageal sphincter pressure is low throughout gestation with a nadir in the third-trimester and returning to normal postpartum.

Many clinical diseases, such as NVP, may be related to gastric dysrhythmia, and slow wave gastric arrhythmias often occur in early pregnancy nausea, affecting 50–70% of pregnant women (95). The abnormal slow wave electrical rhythm of the stomach will inevitably affect peristalsis and emptying of the stomach, thus causing nausea and vomiting (100). In a comparative study, researchers examined the electrogastrography (EGG) readings of eight pregnant and non-pregnant women with nausea and found that five of the women with nausea developed arrhythmias during the first trimester of pregnancy (74). Koch et al. used EGG to confirm that women with normal slow-wave activity had few complaints of nausea during pregnancy (95). Similarly, in a dermatogastriogram study, Riezzo et al. demonstrated that pregnant women without nausea and vomiting had normal myoelectric activity at 3-CPM while performing EGG recording (96). In addition, Koch et al. found that many women with gestational nausea had gastric slow-wave disruption, or gastric tachygastrias, or bradygastrias. Twenty-six of 32 women with nausea in early pregnancy had slow-wave dysrhythmia, 17 had tachygastrias, and four had no gastric electrical activity. Postnatal records showed a return to normal slow wave rhythm and relief of symptoms. In contrast, pregnant women without active nausea and vomiting do not exhibit bradygastrias (74, 95).

While studies suggest a link between gastric dysrhythmia and NVP, future research should focus on repeating these studies in patients with HG. From this it may be possible to conclude whether or not upper intestinal motility disorders are associated with HG.

### Placenta Related Factors

Fetal growth depends on a well-functioning placenta and a suitable environment in the womb, and placental weight has been considered to reflect placental function (101). Several studies have found a link between, for example, the weight of the placenta, the hormones it produces, genes (GDF15, IGFBP7, and PGR) expressed in the placenta, and HG or NVP (1, 30, 101, 102). For example, intact hydatidiform mole (a growth characterized by placental development in which the placental villi trophoblast cells proliferate abnormally in the absence of an embryo) is associated with HG (1).

The relationship between placental function and HG is unclear. In a population-based cohort study of 200,390 pregnant women, placental weight relative to the infant's birth weight (PW/BW ratio) was introduced. Vandraas et al. found that compared with women without HG, HG women with female offspring had a much higher PW/BW ratio, and these women had an almost 20% increased risk of having a PW/BW ratio in the top tenth percentile (101). Fetal sex was found to be a modifying factor in the association between HG and PW/BW ratios, the mechanism of which is still unclear. One possible reason is that high circulating levels of estrogen and hCG, produced primarily by the placenta, may be responsible for HG. Female offspring are associated with increased levels of these hormones as well as increased prevalence and severity of HG (101). Similar to estrogen and hCG, GDF15 was shown to be increased in pregnancies of female offspring in a recent study by Andersson-Hall et al. (103). Furthermore, Gadsby et al. used the total hours of nausea in the first trimester as a continuous variable to report associations with various factors related to a woman's obstetric and personal history. Preliminary analysis showed that women with heavier placentas (Spearman's correlation coefficient, *p* = 0.005) had higher levels of nausea in the prospective study (30).

In contrast, a prospective cohort study of 2,253 pregnant women found that there was no correlation between HG and placental dysfunction. It was also found that the placenta of women with mild vomiting was lighter, while that of women with severe vomiting was heavier. Similarly, the ratio of PW/BW was lower in those with mild vomiting, and higher in those with severe vomiting, but the differences were not statistically significant (104).

Two kinds of tachykinins are found in the placenta, endokinin (EKB) also known as hemokinin and neurokinin A (NKA). They are signaled by three G protein-coupled neurokinin receptors (NK1-3R). Researchers found a significant correlation between the timing of vomiting and changes in the mother's vasculature, suggesting that the placental EKB stimulation system NK1RS may be involved in a healthy pregnancy. Another supportive coincidence is the observation that women who do not experience vomiting during pregnancy presumably have a natural downregulation or reduced sensitivity of the NK1 vomiting receptor in their brains (102).

Placental prostaglandin E2 (PGE2) may play a role in the pathogenesis of NVP owing to its effect on gastric smooth muscle. North et al. quantitatively analyzed serum PGE2 of pregnant women and found that 18 pregnant women with NVP had higher levels of PGE2 than those who had no symptoms (11). While the evidence linking hCG to HG remains inconclusive, it may have important paracrine or autocrine effects on placental prostaglandins, and the maximum stimulation of placental prostaglandin production induced by hCG occurs in the placenta at 9–12 weeks (105).

Cell-free fetal DNA (cff-DNA) is a new and promising biomarker. Recent studies have shown that the main source of cff-DNA is cells from the syncytial trophoblast, from which they are released from syncytium junctions (106). In a clinical trial, Sekizawa et al. found significantly higher concentrations of cff-DNA in 16 women with HG compared to 23 women with normal pregnancies (107). Elevated levels of cff-DNA were also found during placental previa (106). These findings suggest a link between placental dysfunction and HG. More experiments are needed to test this hypothesis by measuring cff-DNA levels and observing pregnancy complications.

### Psychosocial Factors

Pregnant women with psychological disorders are inclined to be exposed to adverse health outcomes, including NVP and HG (48, 108). So far, 17 studies suggest that adverse psychological factors are associated with HG or NVP. Possible adverse psychological factors include depression, anxiety, mood disorders, and stress (109–111). Evidence supporting this theory is discussed below.

In a structured clinical interview, patients with HG had a higher prevalence of mood or anxiety disorders before pregnancy compared with women without HG (32.7% vs. 10.0%) (109). Paid work has also been found to be an independent risk factor for anxiety. Tan et al. conducted a prospective study and reported that 57.4% of HG women met the criteria for depression or anxiety, some met either, and some met both. And a paid job is linked with anxiety (112). Although the paid work may be a risk factor, losing paid work may be a consequence or even worsen the symptoms of HG. Furthermore, a study found that women with HG had significantly higher scores of depression and anxiety, suggesting that the mood and anxiety disorders may be associated with the pathogenesis of HG in pregnant women (113).

Children born to depressed mothers are reported to show increased rates of mood and cognitive problems, such as attention deficit/hyperactivity disorder and anxiety, as well as delayed language skills (114). Therefore, screening for maternal depression is necessary for fetal prognosis. It is unclear whether depression plays a role in the etiology of HG or is a consequence of HG. In a cross-sectional study, it was found that 78.9% of the HG patients had some degree of depression, as opposed to only 5% of the control group (110). Another prospective study showed that in a sample of 47 patients with HG, one third of the women had at least one psychiatric diagnosis, which suggested that psychiatric disorders may play a significant role in the etiology of HG (115). In another study of NVP, the researchers found a positive correlation between the severity of nausea and vomiting and the severity of depression (116).

A cross-sectional and comparative study showed that women with mild nausea and vomiting were less stressed than those with severe nausea and vomiting. Therefore, perceived stress levels and maternal adjustment may be related to the severity of nausea and vomiting during pregnancy. Another study showed that the severity of NVP was significantly associated with obsessive-compulsive disorder (OCD) and alexithymia during the first trimester (117).

In a longitudinal cohort study, the prevalence of nausea, vomiting, anxiety, depression, and stress in newly admitted HG pregnant women was 100, 100, 69, 19, and 21%, respectively, decreasing to 15.7, 9.9, 19, 4, and 3% at the end of pregnancy. In the third trimester, when the HG group was compared to the control group, the risk of nausea or vomiting was similar, but the risk of depression, anxiety, and stress was significantly lower: adjusted odds ratio (AOR) 0.10 (95% CI 0.03–0.5), 0.11 (0.05–0.23), and 0.08 (0.02–0.33), respectively (118). The study revealed a pattern in which women who vomited during pregnancy experienced a strong rebound from depression, anxiety, and stress. By the third trimester, the psychological stress level of women who vomited acutely during pregnancy was even lower than in the control group. This observation suggests that much of the psychological distress of acute HG is self-limiting (118).

In brief, psychological factors may be closely related to HG/NVP. However, the vast majority of women with HG do not have any pre-existing history of a psychological disorder. It is also important to notice that ill patients are significantly more likely to have a lower score on psychological tests than healthy controls, or a lower score assessed prior to or after pregnancy (119). The historical emphasis on a psychiatric etiology has resulted in undertreatment and mistreatment of women with NVP and HG. This should be addressed with appropriate consideration of the caveats and potential for harm (120). Therefore, more well-designed experiments should be conducted with a more cautious approach.

In summary, the above studies have indicated that the etiology of NVP and HG is regulated by multiple factors, in particular the GDF15- GFRAL axis. Focus should now be placed on those factors that can synergize or antagonize the occurrence of NVP and HG.

## Management

### Pharmacological Treatment of HG and NVP

NVP is caused by different mechanisms but most of them are unknown. It is known that combining antiemetics with different mechanisms of action can improve the antiemetic effect during systematic therapy (121). If NVP or HG are left unmanaged, patients with serious cases, whose treatment will be delayed, may suffer needlessly and encounter hospitalization or multiple emergency room visits. Health plans may improve the quality of life and reduce unnecessary treatment through using evidence-based pharmacologic interventions that are known to be effective, safe, and cost effective (122). Therefore, below we summarize pharmacological practice in clinics for NVP or HG.

Ondansetron is a selective 5-HT3 receptor antagonist, that has been approved for the treatment of nausea and vomiting related to cancer chemotherapy, surgery, and pregnancy (123). Previous studies have shown it to be the most prevalent antiemetic drug used for NVP in the USA (124). A meta-analysis and review of ondansetron and the risk of major congenital malformations reported no increased rate of major or selected subgroups of malformations, especially for heart defects or orofacial clefts (125). A clinical trial in Western Australia (including 251 pregnant women) also did not detect any adverse outcomes from the administration of ondansetron in pregnancy (126). Meanwhile, rare adverse effects include a prolonged QT interval and serotonin syndrome (which may include agitation, high body temperature, and increased reflexes) (127). Additionally, Fejzo et al. compared outcomes in 1,841 HG pregnancies exposed and unexposed to ondansetron and found that women who took ondansetron had a notably lower rate of termination of their pregnancies owing to HG and lower rate of spontaneous abortion in the first 12 weeks of pregnancy (128). Consequently, women taking ondansetron were more likely to report a live birth. In 2015, Flake et al. revealed that ondansetron can reduce nausea and vomiting in children with acute gastroenteritis and in women with HG by blocking dopamine in the intestines and chemoreceptor trigger zone (129). Another study reported that ondansetron was superior to the combination of pyridoxine and doxylamine in the treatment of nausea and emesis in pregnancy, based on a randomized controlled trial including 36 pregnant women (130). Similarly, based on the study of Kashifard et al., the incidence of NVP was significantly lower in an ondansetron group than metoclopramide group (pregnant women = 83, and gestational age 8.7 weeks) (131). Additionally, ondansetron was demonstrated to have antiemetic and antinauseant effects in HG; moreover, it had less adverse effects and was less expensive than metoclopramide (131, 132). Meanwhile, the safety of ondansetron during pregnancy was reported in a Danish study of 1970 exposed infants, who did not show an increased risk of fetal malformations or adverse pregnancy outcomes. The study also showed that ondansetron was not associated with an increased risk for major malformations above baseline compared with usual treatment (133), indicating its safety in pregnant women. However, some studies have associated ondansetron with certain birth defects. A Swedish cohort of 1,349 exposed pregnant women found an increased risk of cardiac septal defects (134), and a US cohort also reported an increased risk of cleft palate (135). In addition, a 2-fold increased risk in cardiac malformations was found in another Danish study (136). Future studies should focus on whether this potential for teratogenic risk is greater than the risk of adverse outcomes if HG is not treated.

Pyridoxine, a vitamer of vitamin B6, is considered to be effective for relieving the severity of nausea in early pregnancy (137, 138). Flake et al. recommended pyridoxine with or without doxylamine, and ginger may also be effective for the treatment of mild pregnancy-induced nausea (129). In addition, a combination of pyridoxine and metoclopramide was found to be superior to either monotherapy in the treatment of NVP (139). However, another placebo-controlled study with 92 women showed that the use of oral pyridoxine in conjunction with metoclopramide during inpatient stays and during the 2 weeks after hospital discharge for HG did not improve vomiting frequency or nausea score (140). Cohort studies have also revealed that metoclopramide does not increase the risk of fetal malformations (141).

Promethazine is primarily an antihistaminergic medication, and also acts as a weak dopamine antagonist. It is effective in treating NVP in pregnancy but has significant maternal side effects including dystonia, sedation, and decreased seizure threshold (142). Droperidol combined with diphenhydramine was also found to reduce days in the hospital for HG; no correlations have been made with fetal malformations, though it is associated with QTc prolongation in some pregnant women (143). Another study showed that promethazine and metoclopramide have a similar therapeutic outcome in pregnant women with HG; of note, promethazine has less adverse effects (144). Strikingly, dimenhydrinate was found to be more effective than vitamin B6 in the treatment of nausea and vomiting in early pregnancy based on 140 pregnant women (145). In a 2021 clinical trial, gabapentin was revealed to be more effective than standard-of-care treatment for reducing NVP scores and increasing overall satisfaction and oral nutrition in pregnant women with HG (146). For HG drug treatment, glucocorticoids cannot reduce re-hospitalization rates when compared to placebo treatment (147). Furthermore, glucocorticoids have been associated with a potential increased risk of oral cleft when used in the early first trimester (148). Other therapeutic options for refractory HG cases include transdermal clonidine to reduce symptoms in women who cannot tolerate oral therapies. A randomized placebo-controlled clinical trial with 13 patients using clonidine showed significant decreases in symptoms and reduced the need for enteral or parenteral nutrition (149). A study of 70 patients with HG showed reduced re-hospitalizations with diazepam compared with intravenous fluid only (150).

### Fluid and Nutritional Support

Generally, the severity of NVP can be assessed using the third stage of pregnancy vomiting/nausea unique quantification (PUQE) questionnaire, with a PUQE score ≤ 6 indicating mild NVP, 7–12 indicating moderate NVP, and ≥13 indicating severe NVP. Severe (PUQE score >13) or protracted (>14 d) moderate NVP requires assessment of the patient's general condition, such as weight loss, ketonuria, or dehydration (that is, her signs of HG), and therefore hospitalization should be considered (1). Intravenous fluids and/or parenteral nutrition or tube feeding may be used as an outpatient treatment (44, 151–154). Hyperemesis level prediction (HELP) score may be better than PUQE in assessing severe disease. HELP classified 92% of women reporting “nothing goes or stays down” as severe, but this was only 58% by using PUQE (155).

Intravenous fluid rehydration is usually recommended for patients with HG who have severe dehydration or ketonuria. Rapid maternal hydration usually relieves many symptoms of HG. In addition to hydration, parenteral nutrition and vitamin and mineral replacement/supplementation will help correct any electrolyte imbalance (44). In a systematic review, the researchers found that glucose saline may be associated with better improvement than normal saline in moderate to severe cases (*n* = 222) (156).

If antiemetic medications and fluids are insufficient to reduce nausea and/or vomiting, ketonuria persists, and the patient is unable to improve nutritional intake, additional nutritional therapy should be considered. Tube feeding is preferred when long-term nutritional therapy is required (1, 157). Enteral tube feeding may be given by a gastric tube or a jejunal tube positioned by gastroscopy (94, 154). Vaisman et al. found that nasojejunal intubation feeding can affect the movement of the gastrointestinal tract, thus inhibiting vomiting during pregnancy (94). However, parenteral nutrition for HG in early pregnancy has been rarely reported (158); but some studies have shown that implantation of an endoscopic jejunostomy (PEG-J) tube in pregnant women can also reduce gastric residual volume, thereby reducing the frequency of vomiting (159). In a study by Vaisman et al., 11 pregnant women with HG were given endoscopic feeding through nasojejunointubation. The extent of vomiting was significantly reduced within 48 h of intubation and completely stopped after an average of 5 ± 4 d (range 1–13 d). This demonstrates that endoscopic feeding with nasojejunal intubation can significantly reduce vomiting through affecting the movement of the gastrointestinal tract (94). Gulley et al. reported that continuous small-caliber nasogastric tube gavage increased gastric motility to reduce HG symptoms in 30 patients with HG, demonstrating that continuous small-caliber nasogastric tube gavage was better at controlling nausea than intravenous (IV) therapy, antiemetic drugs, or avoidance of oral intake (158). Moreover, Garg et al. reported on three women with severe HG who underwent endoscopic placement of PEG-J tubes to maintain nutrition, and all had a successful mother-to-child outcome (159). However, one study showed that early enteral feeding did not improve birth weight or secondary outcomes in patients with HG (154). Also, many women discontinue tube feeding owing to discomfort, indicating that tube feeding is poorly tolerated as an early routine treatment for HG (154).

Thiamine (100 ml of 0.9% sodium chloride contains 100 milligrams, this formula is different from most non-prescription drugs thiamine supplements) should be given when parenteral nutrition is initiated to reduce the risk of refeeding syndrome and Wernicke's encephalopathy. For vomiting and/or low food intake for >2 weeks, it is advised to start parenteral therapy before other treatment, including the infusion of 10% glucose (5% solution is not regarded as a nutritional supplement) (1, 152).

### Complementary Treatments

Treatment for NVP begins with non-pharmaceutical methods such as acupuncture, acupressure, and ginger (160); the effectiveness of these treatments has been demonstrated by some researchers (161–164). A meta-analysis suggested that acupuncture was effective in treating HG according to a total of 16 trials covering 1,043 gravidas (165).

PC-6 acupoint is a traditional Chinese medicine point, located 2 cm above the transverse crease of the wrist, between the tendon of palpal longus and the tendon of flexor carpi radialis (166–168) which is believed to be an effective point for the treatment of nausea and vomiting (169). According to the principle of Qi, applying pressure to PC-6 points can slowly block abnormal energy and relieve symptoms related to PC-6 points (170). In addition, a review of seven trials also showed that PC-6 acupuncture points can help with nausea (171). Methods of treating nausea and vomiting in pregnant women by acting on PC-6 acupoint include acupuncture and acupoint pressing (172).

Acupuncture is an effective non-pharmaceutical method of treating HG by inserting needles into the PC-6 acupoint (173). As a traditional Chinese method of treating HG, it can control and coordinate sympathetic and parasympathetic responses to pain, as well as other nerve conduction related to injury and disease, and conduct stimulation through the PC-6 acupoint, to reduce nausea and vomiting symptoms in pregnant women (166). For example, Smith et al. set up a controlled trial with traditional acupuncture (which used groups of acupuncture points on the mid and upper abdomen), PC-6 acupuncture, and no acupuncture, and observed the severity of nausea and the times of vomiting. After 4 weeks, they found that PC-6 acupuncture was more effective in reducing nausea and vomiting in women than traditional acupuncture or no acupuncture (174). Similarly, Sridharan et al. also found that acupuncture reduced nausea and vomiting in pregnant women (161). In addition, in a comparative study of 90 patients, Mao et al. found that after 7 d of treatment, the total effective rate in the acupuncture group was 96.7%, and the experimental results demonstrated that acupuncture was rapid, obvious, and without adverse effects in the treatment of pregnancy vomiting (175). However, in the absence of high-quality randomized trials, the benefits of acupuncture in the treatment of nausea and vomiting in early pregnancy remains skeptical (176). In a study by Knight *et al.*, neither acupuncture or sham acupuncture were effective in treating women with nausea and vomiting during pregnancy (177).

Acupressure is an effective non-pharmaceutical method to relieve nausea and vomiting symptoms by applying pressure at the PC-6 acupoint to stimulate the median nerve (167). Acupressure not only has a potential effect on the nausea of postoperative patients and chemotherapy patients, but also plays an important role in reducing the degree of nausea and vomiting in pregnant women (178). In a 120-person randomized controlled trial comparing the severity of nausea, vomiting, and retching symptoms in pregnant women treated with acupressure vs. placebo, Adlan et al. demonstrated that PC-6 acupressure significantly reduced nausea, vomiting, and retching symptoms (173). In addition, Aloysio et al. demonstrated that PC-6 acupressure was effective in reducing pregnancy nausea and vomiting compared to a placebo in a single-blind, 7-d randomized study of 66 patients (179). Matthews et al. found that acupressure in the NVP group had a similar effect to vitamin B6 in reducing nausea and vomiting during pregnancy, but not in the placebo group (180). However, the mechanism of PC-6 acupressure for HG is unclear, and most women and healthcare professionals remain skeptical of its benefits (173). Heazell et al. observed that P6 acupressure does not decrease the requirement for antiemetic medication, the amount of intravenous fluid, or median duration of hospitalization compared with a placebo (169). Therefore, further clinical studies are needed to evaluate the mode of action and efficacy of acupressure under multi-factor conditions.

Ginger therapy is a simple, easily available, convenient, and effective method for the treatment of nausea and vomiting during pregnancy (176). Ginger helps improve NVP by stimulating the movement of the gastrointestinal tract and the flow of saliva, bile, and gastric secretions. Extracts from ginger not only inhibit the growth of some *H. pylori* strains, but also, one of its components has been shown to have similar activity to the 5-HT3 antagonist ondansetron in reducing nausea and vomiting during pregnancy (181). The safe treatment of 1,000 mg ginger per day for 4 d can improve the symptoms of nausea and vomiting in pregnant women (176), and this effect may be caused by anticholinergic and antihistamine properties (182). In a comparison trial involving 70 patients, Vutyavanich et al. compared changes in the degree of nausea between two groups by using Fisher's exact test, and finally demonstrated that ginger could effectively alleviate the severity of nausea and vomiting during pregnancy without significant side effects (183, 184). In addition, Saberi et al. also showed in a controlled trial that ginger effectively alleviated mild to moderate nausea and vomiting in pregnant women under 16 weeks of gestation (185, 186). In a controlled trial, Backon et al. reported that the use of ginger to reduce the severity of nausea and vomiting during the first 3 months was safe and better than a placebo and pyridoxine (187, 188). However, Portnoi et al. reported that ginger can reduce the severity of general NVP symptoms and nausea, but had no significant effect on vomiting compared with a placebo (189). Strikingly, an internet-based survey of women with HG reported that 87% of respondents had tried ginger to relieve symptoms, and 88% of those reported that it was completely ineffective; 51% of respondents reported that their symptoms exacerbated, and 82% reported that the use of ginger caused a worsening of their mood, such as feelings of anger, lack of validation, isolation, guilt, and exacerbated the feeling that they were misunderstood. In addition, 79% of women who were recommended to take ginger by a health care professional (HCP) reported that it damage their trust and confidence in the HCP (190). More research is needed to demonstrate the efficacy of ginger in the treatment of nausea and vomiting during pregnancy. In addition, owing to the lack of safety studies at doses >1,000 mg/d and because of its potential inhibitory effect on platelet function, ginger is not recommended for pregnant patients receiving anticoagulant therapy (188).

## Discussion

Although NVP and HG are common problems in pregnant women, studies focusing on pathogenesis are lacking. The reason may be that NVP is often considered normal and self-limiting, but the disease burden of NVP and severe HG is largely underestimated. According to recent research, more clinical trials and other studies related to NVP and HG are raising awareness and support for pregnant women, which is critical for further efforts to address this challenge. In 2018, the discovery of the first genome-wide association study (GWAS) of NVP and HG provided novel insights into their etiology by demonstrating that the placenta and appetite hormone gene *GDF15*, is a genetic risk factor (31). This suggests that abnormal gene expression levels may contribute to the etiology of NVP and HG, thus providing a new and promising approach to understanding the pathogenesis at the molecular level. Additionally, we need to focus on whether proteins encoded by *GDF15* can be used for the diagnosis, prediction, and treatment of NVP and HG. Meanwhile, Turco et al. reported an organoid model for placental development, which closely resembles the normal first-trimester placenta; this advance will help elucidate the role of genetic factors in placental biology (191). In addition, drugs targeting the *GDF15*-*GFRAL* axis have also been developed to treat cancer-associated cachexia, which is correlated with high levels of GDF15 (192). Furthermore, targeting *GDF15* can improve body weight and improve multiple metabolic diseases in mice models (193). Another study revealed that the inhibition of *GDF15* restored muscle and appetite to reverse cancer-associated cachexia in animal models (36). Therefore, developing drugs targeting the *GDF15*-*GFRAL* pathway, if proven safe in pregnancy, may help in the treatment of women with NVP or HG.

Currently, only a few antiemetics are approved for the treatment of NVP and HG, however, the success of ondansetron in the USA is gaining increasing attention (124, 194). As many as 20% of pregnant women in the USA are taking ondansetron and increasingly using medical marijuana (195, 196), indicating that the burden of NVP is large and there is a sizable market for antiemetics to treat NVP. However, with the severe clinical outcome of HG, delayed awareness and the delayed seeking of medical help remain important factors that undermine prognosis (157). Importantly, more studies regarding the safety and efficacy of the current treatment strategies for NVP and HG are urgently needed, especially regarding antiemetics exposure and their association with specific structural birth defects in offspring (197). Hence, an international consensus on therapy for NVP and HG is needed (21). It is worth noting that mother and child may have a higher risk from untreated HG compared with standard treatment with the antiemetic drugs. Interestingly, some studies that focused on the same antiemetic drugs produced differing results. For instance, as mentioned earlier, plenty of studies have demonstrated the efficacy and safety of ondansetron (126). However, another study based on a large US commercially-insured population, found that ondansetron exposure was associated with increased risk of cardiac and orofacial cleft defects compared with no antiemetic exposure (196). Meanwhile, routes of administration for antiemetics (patches or suppositories) and nutritional regimens (nutritional supplementation or fluids supplementation) can also affect the outcome of NVP and HG. Overall, new clinical studies must be initiated to determine whether early intervention can stop the progression of NVP to HG through strictly controlled variables.

We can conclude that NVP is a common disease in pregnant women that ranges in spectrum from mild to moderate nausea and vomiting, and HG is a pathologic NVP, contributing to severe clinical outcomes. However, although mounting evidence suggests a role for GDF15, the exact pathogenesis is still largely unclear. Treatment of NVP and HG is mainly symptomatic therapy from dietary changes and oral antiemetic drugs to hospitalization with fluid and nutrition supplements. Most studies suggest that NVP is not harmful to the fetus, but it can significantly reduce quality of life during pregnancy. For women with HG, maternal and fetal morbidity (such as abortion) may occur if HG is unrecognized and not treated in a timely fashion. Overall, more basic studies and clinical trials are required to elucidate the pathogenesis of NVP and HG and develop novel therapeutics for these patients.

### Method and Literature Mining Strategy

We searched for relevant articles in English in the MEDLINE database. Relevant articles were selected against the specific keywords as per the outlines of the study by using the database. In addition to the relevance of the title and abstracts, articles were selected for inclusion based on the topic of the manuscript. The search period was from the date when the database was established to November 2021. In addition, we performed a manual search of related references not in the electronic search. All selected articles have been cited accordingly. The MEDLINE search strategy is presented in Box 1.

Box 1 **The MEDLINE search strategy was summarized in the flow chart.**

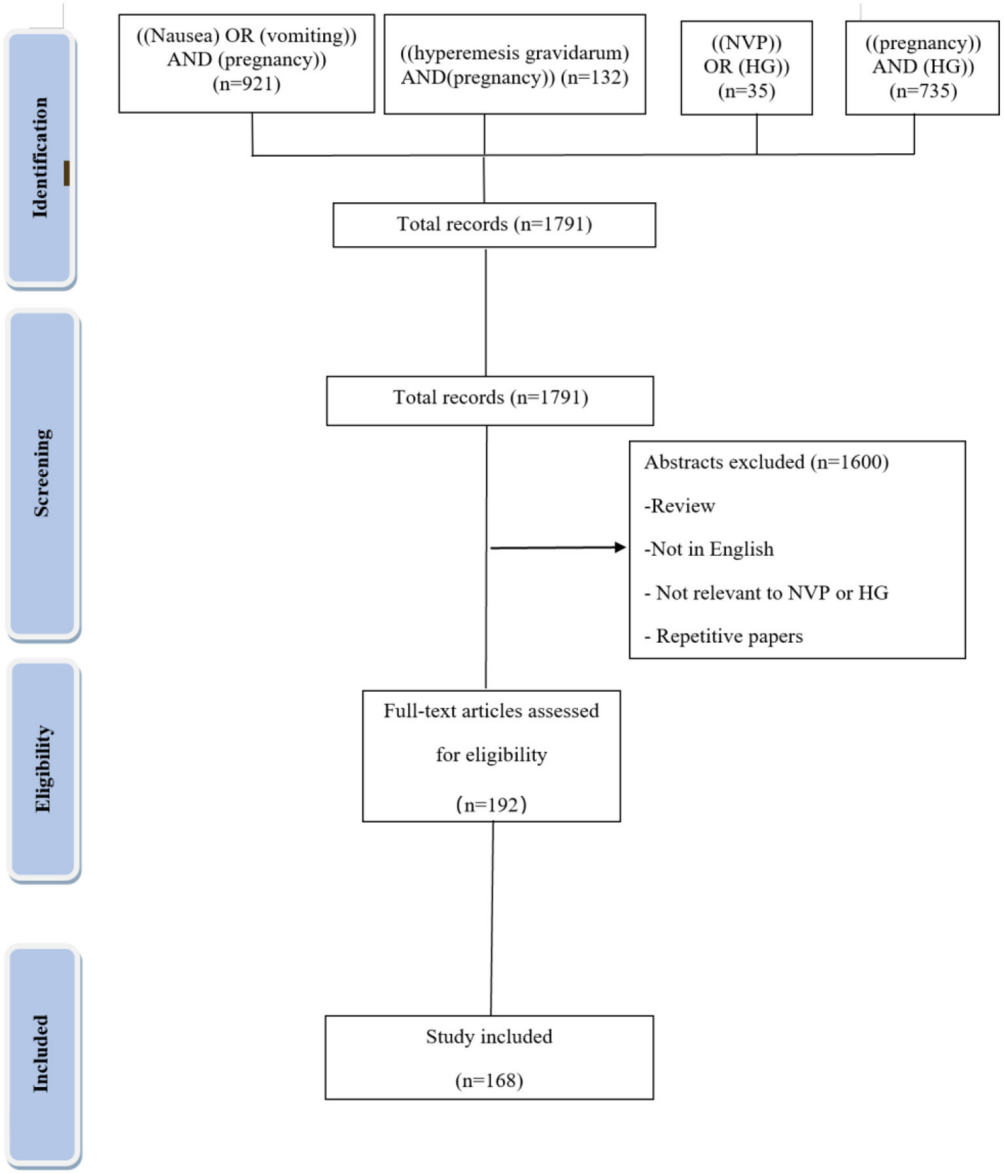

## Author Contributions

CL, GZ, DQ, LW, YH, MZ, and YF reviewed the literature and prepared the manuscript draft and the figure. EJ made the final editing and offered his expert suggestions and insights in preparing this work. All authors have read and agreed to the published version of the manuscript.

## Funding

This review was supported by the Projects for College Students in Henan University (No. 20211022001), National Natural Science Foundation of China (No. 81900375), and Henan Provincial Science and Technology Research Project (No. 212102310147).

## Conflict of Interest

The authors declare that the research was conducted in the absence of any commercial or financial relationships that could be construed as a potential conflict of interest.

## Publisher's Note

All claims expressed in this article are solely those of the authors and do not necessarily represent those of their affiliated organizations, or those of the publisher, the editors and the reviewers. Any product that may be evaluated in this article, or claim that may be made by its manufacturer, is not guaranteed or endorsed by the publisher.
